# Healing touch in radiation therapy: is the benefit tangible?

**DOI:** 10.18632/oncotarget.20594

**Published:** 2017-08-30

**Authors:** Jean-Baptiste Guy, Sacha Bard-Reboul, Jane-Chloé Trone, Alexis Vallard, Sophie Espenel, Julien Langrand-Escure, Anis Hamrouni, Majed Ben Mrad, Stéphanie Morisson, Patrick Michaud, Nicolas Magné, Chloé Rancoule

**Affiliations:** ^1^ Département de Radiothérapie, Institut de Cancérologie de la Loire-Lucien Neuwirth, Saint-Priest-en-Jarez, France; ^2^ Département Interdisciplinaire des Soins de Supports, Institut de Cancérologie de la Loire-Lucien Neuwirth, Saint-Priest-en-Jarez, France

**Keywords:** complementary and alternative medicine (CAM), radiation therapy, supportive care, prostate cancer, breast cancer

## Abstract

**Background:**

Cancer patients tend to use more and more complementary or alternative medicine concomitantly to radiotherapy. A large part of these patients have recourse to Mind and Body practice, mainly with biofield healers or magnetizers, without any level of evidence. The aim of the present study was to report epidemiologic data on biofield healers in radiation therapy patients, and to assess the possible objective and subjective benefits.

**Materials and Methods:**

A retrospective study was conducted in a French cancer institute. All consecutive breast or prostate cancer patients undergoing a curative radiotherapy during 2015 were screened (*n* = 806). Healer consultation procedure, frequency, and remuneration were collected. Patient's self-evaluation of healer's impact on treatment tolerance was reported. Tolerance (fatigue, pain) was assessed through visual analogic scale (0 to 10). Analgesic consumption was evaluated. Toxicities were described according to NTCAEv4.0.

**Results:**

500 patients were included (350 women and 150 men). A total of 256 patients (51.2%) consulted a healer during their radiation treatment, with a majority of women (58%, *p* < 0.01). Most of patients had weekly (*n* = 209, 41.8%) or daily (*n* = 84, 16.8%) appointments with their healer. Regarding the self-reported tolerance, > 80% of the patients described a “good” or “very good” impact of the healer on their treatment. Healers were mainly voluntary (75.8%). Regarding the clinical efficacy, no difference was observed in prostate and in breast cancer patients (toxicity, antalgic consumption, pain).

**Conclusions:**

This study reveals that the majority of patients treated by radiotherapy consults a healer and reports a benefit on subjective tolerance, without objective tolerance amelioration.

## INTRODUCTION

In spite of the supportive care progresses, cancer treatments are still associated with high rates of side effects, limiting patient's quality of life [1]. Contrasting with this evidence-based medicine development, cancer patients are reported to increasingly use Complementary or Alternative Medicine (CAM) [2–4]. According to the National Center for Complementary and Integrative Health (NCCIH), CAMs are defined as practices, health care systems or products that cannot be considered as conventional medicine. The frequency of CAM use is variable regarding studies with reported rates of 30–50%, mainly depending on cancer locations [5–7], type of treatments, and countries [8]. Mind and Body practices are part of CAM and are frequently used by cancer patients. These practices include diverse procedures, with yoga, chiropractic manipulation, meditation but also healing or therapeutic touch (HT), distant healing, or Reiki therapy [9, 10].

In French daily routine, radiotherapy patients often fear radiation-induced dermatitis and frequently see distant healers, also called “biofield healers”. Biofield healers are believed to be able to prevent and limit radiation dermatitis through hand imposition, therapeutic or healing touch, breathe and healing symbols. Although radiotherapy patients frequently report to see biofield healers in daily routine practice, the rate of utilization, the cost, the efficacy and the nature of the healer procedure has only been very poorly described [11].

The main objective of the present study was to collect data on practices and frequency of use of biofield healers in radiotherapy patients. The secondary objective was to evaluate the subjective and objective benefit of biofield healers on tolerance to treatment and on toxicity

## MATERIALS AND METHODS

### Study design

**Figure 1:**
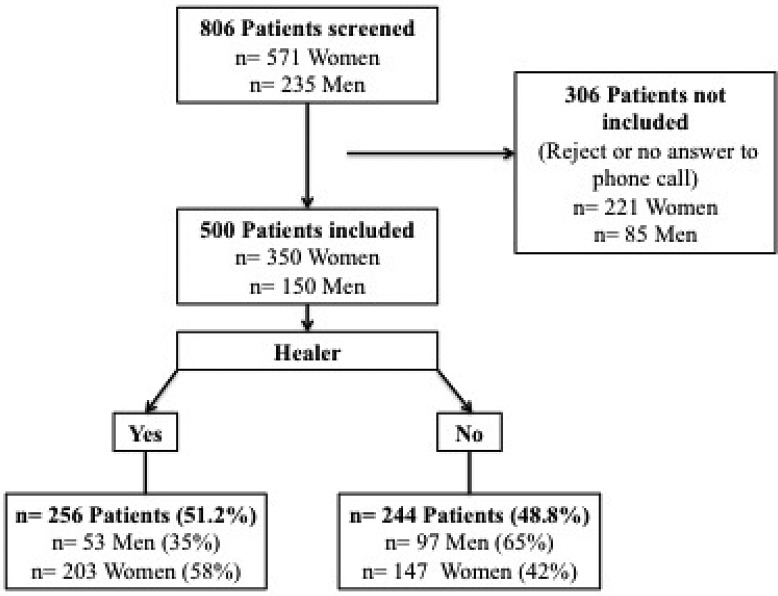
Study Flow Chart n: number of patients.

A retrospective survey was conducted at the Lucien Neuwirth Cancer Institute, based on a questionnaire (completed by the patient), and on clinical data (collected in medical records by radiation oncologists). All women undergoing a radiotherapy for a breast cancer in 2015 in the department were screened. All men treated with radiation therapy for a prostate cancer in the department in 2015 were screened. The flow chart summarizes the patient selection (Figure 1). The institutional ethics committee approved the study, which was conducted in compliance with the Helsinki Declaration.

### Study endpoints

Patients were consecutively phoned at the end of their radiation treatment, and answered the questionnaire on biofield healers (Annex 1). Patient's consent was obtained before each questionnaire completion. Phone conversations lasted approximately for five minutes.

Collected data were: healer appointment, Yes/No; Frequency; healer procedure: Appointment/Phone/Picture; healer technique or action: Hand imposition (Therapeutic or Healing Touch)/Breathe/Healing Symbols; Remuneration; Global tolerance of treatment: 0 to 10 scale (0 = Bad tolerance; 10 = Excellent tolerance); Pain evaluation: visual analogic scale (VAS, 0 = No pain; 10= Maximal pain imaginable); Analgesics consumption; Fatigue hetero-evaluation: scale from 0 to 10 (0 = No fatigue; 10 = Extreme fatigue). Self-evaluation by patient of healer's impact on treatment tolerance was also reported (Impact: None, A little, Good or Very Good).

A second step consisted in a retrospective analysis of patient clinical characteristics and toxicities based on medical records. Data were collected in electronic medical records by a blinded radiation oncologist. Collected data were: Age; Weight; Height; Body Mass Index (BMI); ECOG Performance Status; Diabetes, Yes/No; Hypertension, Yes/No; Tobacco consuming; Chemotherapy, Yes/No; Cancer histology and TNM classification. Technical characteristics on RT Treatment (Dose/Fields) were reported, and toxicities were collected according to Common Terminology Classification for Adverse Events (CTCAE) v 4.0: skin toxicity for women, urinary toxicity for men.

### Statistical analysis

Chi-squared test was performed to evaluate differences between groups with or without healers. Z test was performed to evaluated difference between mean values (all patient sets were > 30). A result was found statistically significant at *p* ≤ 0.05 (the symbol * underlines a significant result). Analyses were performed on R software [12].

## RESULTS

Among the 806 patients screened for this study, 500 patients were included: 350 women and 150 Men. 306 patients could not be included due to refusal or no answer to phone call (three attempts). The flow chart (Figure 1) summarizes patient's inclusion.

### Clinical characteristics

The mean age was 65.4 years, with 70.0 years for men and 63.3 years for women. The mean Body Mass Index (BMI) was 26.3 Kgs/m^2^, with 27.2 for men and 26.1 for women. 27.4% of the patients had blood hypertension, and 8.2% were active smokers. Repartition of sex, chemotherapy, or endocrine therapy was not statistically different in the two groups of patients (seeing a biofield healer versus not seeing a biofield healer). Patient characteristics are reported in Table 1. A large majority of women received a conventionally fractionated radiation scheme, with 2 Gy per fraction for a total dose of 50 Gy in 25 daily fractions, with a subsequent radiation boost of 10–16 Gy to the tumor bed. Prostate cancer radiation doses varied with to the radiation setting, from 66 Gy for post-operative radiotherapy to 78–80 Gy for curative exclusive radiotherapy (Tables 4 and 5).

**Table 1:** Clinical characteristics of patients

	With Healer 256 (51.2%)	Without Healer 244 (48.8%)	All 500 (100%)	*p*-value
**Men**	53 (35%)	97 (65%)	150	*p* < 0.01*
**Women**	203 (58%)	147 (42%)	350	
***Clinical characteristics***				
**Age (Years)**_*Mean ± Stand Dev*_	64.1 ± 11.1	66.6 ± 11	65.35 ± 11.1	*p* = 0.01*
Women	62.6 ± 11.6	64.3 ± 12.7	63.45 ± 12.1	*p* = 0.62
Men	69.9 ± 5.7	70.1 ± 6.9	70.0 ± 6.2	*p* = 0.86
**Weight (Kgs)**_***Mean ± Stand Dev***_	69.4 ± 16.4	72.4 ± 14.2	70.9 ± 15.3	*p* = 0.03*
Women	67.0 ± 13.7	67.8 ± 15.2	67.5 ± 14.5	*p* = 0.99
Men	81.5 ± 10.4	81.1 ± 15.1	81.3 ± 12.3	*p* = 0.83
**Height (m)**_*Mean ± Stand Dev*_	1.64 ± 0.08	1.65 ± 0.08	1.65 ± 0.08	*p* = 0.23
Women	1.62 ± 0.06	1.61 ± 0.06	1.61 ± 0.06	*p* = 0.06
Men	1.73 ± 0.06	1.72 ± 0.07	1.73 ± 0.07	*p* = 0.22
**BMI (Kgs/m^2^)**_*Mean ± Stand Dev*_	25.95 ± 5.4	26.55 ± 4.9	26.25 ± 5.1	*p* = 0.17
Women	26.1 ± 5.1	26.1 ± 5.9	26.1 ± 5.4	*p* = 0.67
Men	27.1 ± 3.3	27.3 ± 4.2	27.2 ± 3.7	*p* = 0.74
***Comorbidities***				
**Diabetes**	12 (4.7%)	17 (7%)	29 (5.8%)	*p* = 0.27
Women	7 (3.4%)	8 (5.4%)	15 (4.3%)	*p* = 0.36
Men	5 (9.4%)	9 (9.3%)	14 (9.3%)	*p* = 0.98
**Hypertension**	70 (27.3%)	67 (27.5%)	137 (27.4%)	*p* = 0.97
Women	60 (29.5%)	40 (27.2%)	100 (28.6%)	*p* = 0.63
Men	10 (18.9%)	27 (27.8%)	37 (24.7%)	*p* = 0.22
**Active smokers**	19 (7.4%)	22 (9%)	41 (8.2%)	*p* = 0.52
Women	18 (8.9%)	14 (9.5%)	32 (9.1%)	*p* = 0.83
Men	1 (1.9%)	8 (8.2%)	9 (6.0%)	-
***Cancer and systemic treatment***				
**Breast Cancer**	203 (58%)	147 (42%)	350 (70%)	
Chemotherapy	86 (42%)	54 (36.7%)	140 (40%)	*p* = 0.24
Hormonal Therapy	1 (0.4%)	3 (1.2%)	4 (1.1%)	-
**Prostate Cancer**	53 (35%)	97 (65%)	150 (30%)	
Chemotherapy	1 (1.9%)	3 (3%)	4 (2.6%)	-
Hormonal Therapy	23 (43.3%)	46 (47.4%)	69 (46%)	*p* = 0.56

Legend: Kgs, Kilograms; m, meters; BMI, Body Mass Index; *, Statistically significant result.

### Biofield healers’ and patients’ practice

Among the 500 patients included in the study, 256 (51.2%) saw a biofield healer during their radiotherapy (Table 1). Patients turning to a biofield healer were mainly females (*n* = 203, 58%, *p* < 0.01). The majority of patients meet the healer at least weekly (41.8%), or daily (16.8%) (Table 2). Biofield healers received patients during a medical appointment is most of the cases (77%). The most reported procedure was hand imposition (42.6%) and breathe (41.8%). Most of the practitioners were unpaid/volunteer (75.8%). No statistical difference was observed between men and women regarding frequency, procedure, action or honorary.

**Table 2:** Characteristics of healers

		Men *n* = 53	Women *n* = 203	All *n* = 256	*p*-value
**Frequency**	Daily	12 (22%)	31 (15%)	43 (16.8%)	*p* = 0.20
	Twice a week	0	15 (7%)	15 (5.8%)	-
	Weekly	23 (43%)	84 (41%)	107 (41.8%)	*p* = 0.79
	2–3 times during treatment	14 (26%)	51 (25%)	65 (25.4%)	*p* = 0.84
	Once during treatment	4 (7%)	23 (11%)	27 (10.5%)	*p* = 0.42
**Procedure**	Medical Appointment	37 (70%)	160 (79%)	197 (77%)	*p* = 0.17
	Phone	12 (23%)	44 (22%)	56 (21.9%)	*p* = 0.88
	Picture	4 (7%)	7 (3%)	11 (4.3%)	-
**Action**	Hand Imposition (Therapeutic or Healing Touch)	17 (32%)	92 (45%)	109 (42.6%)	*p* = 0.08
	Breathe	23 (43%)	84 (41%)	107 (41.8%)	*p* = 0.79
	Healing Symbols	6 (11%)	19 (9%)	25 (9.8%)	*p* = 0.67
	Unknown Action	12 (23%)	24 (12%)	36 (14.1%)	*p* = 0.043*
**Gift**	None	45 (85%)	149 (73%)	194 (75.8%)	*p* = 0.08
	< 50 €	3 (6%)	26 (13%)	29 (11.3%)	*p* = 0.14
	Between 50 and 100 €	3 (6%)	16 (8%)	19 (7.4%)	*p* = 0.58
	> 100 €	1 (2%)	6 (3%)	7 (2.7%)	-

Legend: *, Statistically significant result.

### Healers’ subjective and objective benefits

Most of patients (*n* = 215, 84%) reported a good or very good impact of the biofield healer on their tolerance to radiotherapy (Table 3). Global tolerance, pain evaluation or analgesics consumption were not statistically different between the patients seeing a biofield healer and the patients not seeing a biofield healer. However, pain and antalgics consumption were extremely low in each group, since most of patients had a VAS < 3 and did not take any analgesics. Fatigue was moderate in women (4.7 and 4.3) and low in men (2.9 and 2.2), with no statistical difference between groups.

**Table 3:** Patient's feeling on healer action and impact on treatment

Healer Impact	Men *n* = 53	Women *n* = 203	All *n* = 256
None	3 (5.6 %)	14 (6.8 %)	17 (6.6%)
A little	7 (13.2 %)	8 (3.9 %)	15 (5.8%)
Good	19 (35.8 %)	68 (33.4 %)	87 (34%)
Very Good	21 (39.6 %)	107 (52.7 %)	128 (50%)

Regarding objective toxicities, no difference was reported regarding radio-induced urinary toxicities in prostate cancer patients. No difference was reported either regarding skin toxicity in breast cancer patients. Grade 1 skin toxicity (faint erythema or dry desquamation of the skin, based on the CTCAEv4.0) was decreased in women seeing a biofield healer (27% *vs* 37%), without reaching a statistical difference (*p* = 0.1). The impact of biofield healer on subjective and objective tolerance to radiation is reported in Tables 3, 4 and 5.

**Table 4:** Treatment characteristics and tolerance according to each group for men (with or without healer)

	Healers - Men	YES *n* = 53	NO *n* = 97	*p*-value
**Radiation Doses**	< 66 Gy	9 (17%)	21 (22%)	*p* = 0.49
	66 Gy	13 (24%)	24 (25%)	*p* = 0.98
	72–74 Gy	17 (32%)	28 (29%)	*p* = 0.68
	78–80 Gy	12 (23%)	19 (20%)	*p* = 0.66
	> 80 Gy	0	1 (1%)	-
**Urinary Toxicity**	Grade 1	31 (62%)	49 (51%)	*p* = 0.49
	Grade 2	11 (17%)	16 (16%)	*p* = 0.71
	Grade 3	0 (0%)	2 (2%)	-
	Grade 4	0 (0%)	2 (2%)	-
	Not Reported	4 (7.5%)	9 (9%)	-
**Global Tolerance (From 0 to 10)**_*Mean*_		7.68	8.17	*p* = 0.11
**Pain Evaluation (From 0 to 10)**_*Mean*_		1.38	0.69	*p* = 0.06
**Antalgics**_*Mean*_		0.038	0.032	*p* = 0.8
**Fatigue (From 0 to 10)**_*Mean*_		2.92	2.19	*p* = 0.12

Legend: Gy, Gray; *, Statistically significant result.

**Table 5:** Treatment characteristics and tolerance according to each group for women (with or without healer)

	Healers - Women	YES *n* = 203	NO *n* = 147	*p*-value
**Radiation Doses Breast Dose (Boost)**	50 (16) Gy	128 (63%)	86 (59%)	*p* = 0.69
	50 (10) Gy	14 (7%)	14 (9%)	*p* = 0.37
	40 Gy	10 (5%)	8 (5%)	*p* = 0.92
**Skin Toxicity**	Grade 1	54 (27%)	54 (37%)	*p* = 0.1
	Grade 2	82 (40%)	57 (39%)	*p* = 0.99
	Grade 3	17 (8%)	9 (6%)	*p* = 0.98
	Grade 4	0 (0%)	1 (0.6%)	-
**Global Tolerance (From 0 to 10)**_*Mean*_		7.14	7.28	*p* = 0.45
**Pain Evaluation (From 0 to 10)**_*Mean*_		2.84	2.36	*p* = 0.11
**Antalgics**_*Mean*_		0.13	0.12	*p* = 0.68
**Fatigue (From 0 to 10)**_***Mean***_		4.74	4.27	*p* = 0.12

Legend: Gy, Gray; *, Statistically significant result.

## DISCUSSION

The present study reports for the first time precise and specific data on biofield healers in a large cohort of French radiotherapy cancer patients. We confirm that radiotherapy patients frequently resort to CAM, and especially turn to biofield healers. In the present study, nearly 50% of prostate and breast cancer patients saw a biofield healer during their radiation therapy. Our results corroborate previous data reporting that patients commonly used CAMs during their cancer treatments [4]. A survey conducted in prostate cancer patients in 2012 reported that 51% of patients used of at least one CAM during their treatment. Nearly a third (26%) even used two different CAMs [5]. In the breast cancer area, it was reported that more than 28% of patients used CAM, which was far bellow the proportions observed in our study [6].

Furthermore, the present study reports biofield healer's procedures, with sometimes surprising results. The use of breath as a healing technique has never been described in literature, although it accounted for more than half of our patients’ set. Therapeutic touch or healing touch technique was the most reported technique in the literature [9] and was found here in only 42% of cases. This technique is close to “Reiki” technique, that was successfully tested in prostate cancer, especially with positive effects on anxious patients [13]. Moreover, this study reveals that this practice is time-consuming for patients, with weekly or daily visits, that was initially thought to possibly cause an increased fatigue. Finally, this practice mostly remained cost-free, with a vast majority of volunteer biofield healer (75%), and therefore did not increase health costs for patients [14].

The analysis and the evaluation of benefits are challenging, with a clear discrepancy between a very positive feedback of patients seeing a biofield healer and an absence of objective tolerance improvement. Subjective benefit of biofield healer was major, with 84 % of patients having a good or very good opinion on the healer's impact on their tolerance to radiotherapy. Yet, our results did not show any significant difference in favor of healers regarding radiation toxicities, related fatigue or pain, corroborating previous data. Thus, a randomized placebo-controlled study evaluating the benefit of healing touch on fatigue in 41 breast cancer patients undergoing radiation therapy failed to prove its positive effect [15]. Regarding the pathophysiological explanations, various hypotheses can be found in literature to rationalize the effect of these practices [16]. One of the explanation might be found in a placebo effect, that was previously reported to decrease the pain and fatigue in radiotherapy patients [17–20]. Althought a benefit was not reported regarding objective clinical toxicities in the present study, the frequency of this practice and the differences of perception between patients and radiation oncologists are major messages for both populations. Furthermore, healing touch was previously reported to have a positive effect on symptoms of chronic fatigue [21], and could therefore still have a positive impact on patient's quality of life.

Our study has several limitations due to the retrospective design. First, among the 806 screened patients, only 500 were finally included. These patients more likely report their subjective benefit on biofield healing, and could overestimate benefits in the healer group. Moreover, practices were highly variable from a healer to another. In parallel, toxicities evaluation might have been biased due to retrospective analysis based on medical patient record. Medical studies on CAM benefit often failed to reach significant due to such inevitable bias [22]. Retrospective analysis of pain or fatigue is highly debatable indeed, and should ideally be explored prospectively.

To conclude, our study reveals that a majority of radiotherapy patients see a biofield healer during their treatment. Data revealed that men and women equally used biofield healers, with at least weakly visits. Althought the objective benefit did not appear significant, the subjective-evaluation demonstrated a great satisfaction of patients, with cost-free practices. At the time of modern radiation therapy era, where patient's quality of life is one of the objective to achieve, biofield healers could help both patient and radiation oncologist. A prospective evaluation including a quality of life evaluation is necessary.
